# Mental Health Outreach via Supportive Text Messages during the COVID-19 Pandemic: Improved Mental Health and Reduced Suicidal Ideation after Six Weeks in Subscribers of Text4Hope Compared to a Control Population

**DOI:** 10.3390/ijerph18042157

**Published:** 2021-02-23

**Authors:** Vincent I. O. Agyapong, Reham Shalaby, Marianne Hrabok, Wesley Vuong, Jasmine M. Noble, April Gusnowski, Kelly Mrklas, Daniel Li, Mark Snaterse, Shireen Surood, Bo Cao, Xin-Min Li, Russell Greiner, Andrew J. Greenshaw

**Affiliations:** 1 Department of Psychiatry, Faculty of Medicine and Dentistry, University of Alberta, Edmonton, AB T6G 2B7, Canada; rshalaby@ualberta.ca (R.S.); marianne.hrabok1@ucalgary.ca (M.H.); jmbrown1@ualberta.ca (J.M.N.); Daniel.Li@albertahealthservices.ca (D.L.); cloudbocao@gmail.com (B.C.); Xinmin@ualberta.ca (X.-M.L.); rgreiner@ualberta.ca (R.G.); andy.greenshaw@ualberta.ca (A.J.G.); 2 Addiction and Mental Health, Alberta Health Services, Edmonton, AB T6G 2B7, Canada; Wesley.Vuong@albertahealthservices.ca (W.V.); April.Gusnowski@albertahealthservices.ca (A.G.); Mark.Snaterse@albertahealthservices.ca (M.S.); shireen.surood@albertahealthservices.ca (S.S.); 3 Cumming School of Medicine, University of Calgary, Calgary, AB T2N 4N1, Canada; 4 Institute of Health Economics, 1200 10405 Jasper Avenue Edmonton, AB T5J 3N4, Canada; 5 Strategic Clinical NetworksTM, Provincial Clinical Excellence, Alberta Health Services, Calgary, AB T2P 2M5, Canada; Kelly.Mrklas@albertahealthservices.ca; 6 Department of Community Health Sciences, Cumming School of Medicine, University of Calgary, Calgary, AB T2N 4N1, Canada; 7 Department of Computing Science, Faculty of Science, University of Alberta, Edmonton, AB T6G 2B7, Canada; 8 Alberta Machine Intelligence Institute, Edmonton, AB T6G 2B7, Canada; 9 Asia-Pacific Economic Cooperation (APEC) Digital Hub for Mental Health, Canada

**Keywords:** COVID-19, pandemic, Text4Hope, text messaging, stress, anxiety, depression

## Abstract

**Background:** In March 2020, Alberta Health Services launched Text4Hope, a free mental health text-message service. The service aimed to alleviate pandemic-associated stress, generalized anxiety disorder (GAD), major depressive disorder (MDD), and suicidal propensity. The effectiveness of Text4Hope was evaluated by comparing psychiatric parameters between two subscriber groups. **Methods:** A comparative cross-sectional study with two arms: Text4Hope subscribers who received daily texts for six weeks, the intervention group (IG); and new Text4Hope subscribers who were yet to receive messages, the control group (CG). Logistic regression models were used in the analysis. **Results:** Participants in the IG had lower prevalence rates for moderate/high stress (78.8% vs. 88.0%), likely GAD (31.4% vs. 46.5%), and likely MDD (36.8% vs. 52.1%), respectively, compared to respondents in the CG. After controlling for demographic variables, the IG remained less likely to self-report symptoms of moderate/high stress (OR = 0.56; 95% CI = 0.41–0.75), likely GAD (OR = 0.55; 95% CI = 0.44–0.68), and likely MDD (OR = 0.50; 95% CI = 0.47–0.73). The mean Composite Mental Health score, the sum of mean scores on the PSS, GAD-7, and PHQ-9 was 20.9% higher in the CG. **Conclusions:** Text4Hope is an effective population-level intervention that helps reduce stress, anxiety, depression, and suicidal thoughts during the COVID-19 pandemic. Similar texting services should be implemented during global crises.

## 1. Introduction

On 11 March 2020, the World Health Organization (WHO) declared Coronavirus disease (COVID-19) to be a global pandemic and public health threat [1]. As of 4 August 2020, there were 18,142,718 confirmed COVID-19 cases worldwide, including 691,013 COVID-19-related deaths [2]. At the same time, Canada reported 116,884 COVID-19 cases and 8945 COVID-19-related deaths [2]. Many governments declared stringent restrictions to contain the spread of COVID-19, with a reported transmissibility rate exceeding other similar viruses [3]. Non-essential businesses and recreational facilities were closed, flights and air traffic stopped, and home schooling replaced in-person instruction. Many governments provided personal instructions, including frequent hand washing, physical distancing, self-isolation/quarantine, and mask wearing [4]. These factors apparently sparked higher levels of anxiety, depression, and suicidal thoughts. Some studies reported mental health deterioration in almost half of study populations, with over 70% worried about personal infection. These figures were more prominent among younger individuals, females, nursing staff, and those needing quarantine or forced to stay at home [5,6]. Long-term effects of COVID-19 are yet to be fully realized and researchers have emphasized a need to incorporate web-based mental health services into healthcare [7]. The pandemic provided a stimulus to integrate technology-based health supports, including mobile-based services like text messaging (TextM). TextM does not compromise public health requirements for physical distancing, while allowing users to receive important mental health support. Moreover, TextM is remotely delivered, scalable, economically reliable, convenient, and there is growing evidence of applicability in mental health [8]. Most Canadians (90%) own mobile phones [9], and as TextM is included in most personal phone plans, TextM delivery is economically feasible for healthcare providers [8]. Generally, text messages are popular [10], and their clinical advantages are well documented as an intervention for alcohol dependence, substance use disorder, and affective disorders [11]. For example, in three separate randomized controlled trials in Ireland and Canada, patients with Major Depressive Disorder recorded less depressive symptoms on standardized self-reported instruments after daily supportive text messages for three months as part of their usual treatment, compared to patients who received only the usual care [12,13,14]. High user satisfaction has also been reported with supportive text message interventions in both in a clinical trial and in a population level program [10,15]. As such, the study team and stakeholders wanted to incorporate supportive text messages as a way to alleviate mental health symptoms in the general population during the COVID-19 pandemic. In March 2020, Alberta Health Services (AHS) inaugurated the Text4Hope TextM service to support mental health of Albertans during COVID-19 [16]. Thousands of people signed up for the service only days after launch and enrollment continues to increase to date. The Text4Hope program sends once daily supportive text messages to subscribers for three consecutive months. Messages were designed in the framework of cognitive behavioral therapy (CBT). Text4Hope aimed to improve psychological resilience, alleviate mental health burdens, and enable development of coping mechanisms to prevent and overcome the potential distress and anxiety that may arise in the COVID-19 pandemic [17]. Participants receive a different non-personalized pre-programmed message from a web application each day for three months. Examples of the messages sent were:

- When bad things happen that we can’t control, we often focus on the things we can’t change. Focus on what you can control; what can you do to help yourself (or someone else) today?
- Put yourself on a media diet. It’s important to stay informed, but only check the news and social media intermittently, rather than continuously.
- Advocate for your needs using assertiveness. Assertiveness is being respectful to you and the other person. Be direct, non-aggressive, and specific with your request.

Canadians seeking mental health support were invited to join the three-month program by texting “COVID19HOPE” to a short code number.

The objective of this study was to assess the effectiveness of Text4Hope in reducing psychological impacts due to COVID-19, six weeks into the service. The study examined subscriber responses, collected between 26 April and 12 July 2020, compared to subscribers who had subscribed earlier and already received messages for six weeks with a matching control group of subscribers who had just started Text4Hope and had not yet received messages. Assessment was completed by comparing scores of psychiatric scales for stress, anxiety, major depressive disorder, suicidal ideation/thoughts of self-harm, and sleep disorder.

## 2. Materials and Methods

**Figure 1:**
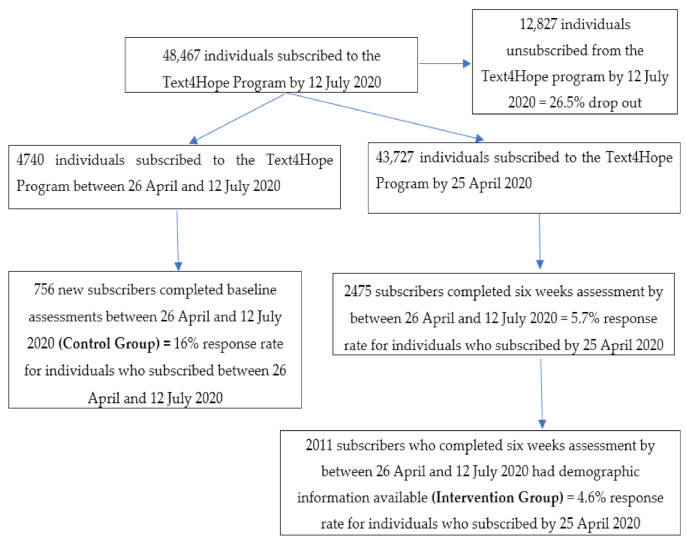
Subscriber flowchart as of 12 July 2020.

We used a comparative cross-sectional survey design involving two study arms of Text4Hope subscribers. Participants in the intervention group (IG) were Text4Hope subscribers who received once daily supportive text messages for six weeks and completed six-week evaluation measures between 26 April and 12 July 2020. Participants in the control group (CG) were Text4Hope subscribers who joined the program in the same time frame and completed baseline evaluation measures before receiving any intervention. Figure 1 shows the number of the participants in the study flow chart.

## 3. Recruitment

Recruitment strategies for this study were as described in the published study protocol [17] and in related publications [18,19,20]. In brief, Text4Hope was launched through an announcement by Alberta’s Chief Medical Officer of Health on behalf of AHS and the Government of Alberta on 23 March 2020. The announcement was broadcast widely across many electronic and print media networks in Alberta to inform Albertans about the program [19]. Additionally, Albertans were made aware of the program via websites dedicated to the service, electronic media, social media feeds, posters at addiction and mental health clinics, emergency departments and wards, and through word of mouth. Subscription to Text4Hope triggered a welcome text message containing a 10-min online survey link requesting demographic characteristics (i.e., gender, age, ethnicity, education, relationship status, employment status, and housing status) and clinical characteristics (self-reported perceived symptoms of stress, anxiety, and depression). Subscribers were only sent a single text message with a survey link each time they were eligible to complete a survey. Clinical characteristics were assessed using validated screening scales for self-reported symptoms, including the Perceived Stress Scale (PSS) (for moderate/high stress; PSS ≥ 14) [21], the Generalized Anxiety Disorder 7-item (GAD-7) scale (for likely generalized anxiety disorder or GAD; GAD-7 ≥ 10) [22], and the Patient Health Questionnaire-9 (PHQ-9) (for likely major depressive disorder or MDD; PHQ-9 ≥ 10) [23]. The PSS is a validated 10-item questionnaire (with an associated Cronbach’s alpha of > 0.70) which is used to assess the self-reported level of stress in the previous 1 month by assessing thoughts and feelings. Each item on the scale is scored between 0 (never) to 5 (very often). Higher scores on the scale indicate higher levels of stress [21]. The GAD-7 is a validated 7-item questionnaire (associated with a Cronbach’s alpha of 0.92) which is used to assess the self-reported levels of anxiety in respondents in the two weeks prior to assessment. Each item on the scale is scored between 0 (not at all) to 4 (nearly everyday). Higher scores on the scale indicate higher levels of anxiety [22]. The PHQ-9 is a 9-item validated instrument (associated with a Cronbach’s alpha of 0.89) which used to diagnose and measure the severity of depression in general medical and mental health settings. Each of the 9 questionnaire items is scored between 0 (not at all) to 3 (nearly every day). Higher scores on the scale indicate higher levels of depression [23]. Since the PHQ-9 asks respondents to reflect on their experience in the past two weeks, we were able to assess recent sleep and suicidal thinking. Specifically, scale items 3 and 9 were used to assess sleep and suicide, respectively. These scales are not formal diagnostic tools. Participant consent was implied via submission of subscribers’ survey responses and a follow-up survey was sent to subscribers six weeks after enrolment in the program. Ethical approval for the research study was obtained through the University of Alberta Health Research Ethics Board (Pro00086163).

## 4. Outcome Measures

For each participant in the CG, we computed individual baseline mean scores on the PSS, GAD-7, and PHQ-9 scales and defined the Composite Mental Health (CMH) score as the sum of these three values. In the same time frame, we computed six-week scores on the PSS, GAD-7, and PHQ-9 scales as well as the CMH score for each IG participant. One primary outcome of this study was the difference of CMH score in the IG, minus the CMH score over the CG subjects. Other primary outcomes were: differences between IG and CG in self-reported prevalence rates of moderate/high stress, likely GAD, and likely MDD. Secondary outcomes were: differences in self-reported rates for disturbed sleep and suicidal ideation/thoughts of self-harm between IG and CG as measured with PHQ-9 scale questions 3 and 9, respectively.

## 5. Hypothesis

We hypothesized that participants receiving daily supportive TextMs for six weeks (IG) would have at least 25% lower CMH scores, and respective prevalence rates for each of moderate/high stress, likely GAD, likely MDD, disturbed sleep, and suicidal ideation/thoughts of self-harm, compared to Text4Hope subscribers who had not yet received the intervention (CG).

## 6. Sample Size Considerations

We estimated a sample size of 62 per group would be sufficient to detect a 25% difference in mean CMH score between the IG and the CG, given a two-sided significance level α = 0.05 and a power of 80% (β = 0.2).

## 7. Analysis

We analyzed the data using IBM Statistical Package for Social Sciences (SPSS) Statistics for Windows, version 26 (IBM Corp., Armonk, NY, USA) [24]. Demographic characteristics and prevalence rates for moderate/high stress, likely GAD, and likely MDD for respondents in both the IG and CG were summarized by numbers and percentages and compared by chi-square analysis with a two tailed criterion (α < 0.05) used to determine statistical differences between the IG and CG (intervention arms) for PSS, GAD-7, and PHQ-9 scale mean scores. In addition, mean CMH scores were compared using independent *t*-tests. Bonferroni correction of the *p*-value was used.

To assess the impact of the supportive text message intervention on our clinical measures, while controlling for demographic characteristics, we entered all demographic predictors along with “intervention arm” into a logistic regression model. Correlation analyses were performed before the logistic regression analysis to rule out very strong correlations among predictor variables. We examined the odds ratios from the binary logistic regression analysis to determine the respective associations between “intervention arm” and the likelihood of respondents self-reporting symptoms of: moderate/high stress, likely GAD, likely MDD, disturbed sleep, and suicidal ideation/thoughts of self-harm in the preceding two weeks, controlling for the other variables in the model. There were no imputations for missing values.

## 8. Results

Table 1 summarizes the demographic characteristics of respondents in both the IG and CG in absolute numbers and percentages. The data in this table indicate that most respondents identified as female (*n* = 2347, 88.0%), aged between 26 and 60 years (*n* = 2036, 76.7%), Caucasian (*n* = 2198, 82.8%), had post-secondary education (*n* = 2054, 87.7%), were married, cohabiting, or partnered (*n* = 1553, 66.3%), employed (*n* = 1638, 70.4%), and lived in their own homes (*n* = 1560, 67.5%). Table 1 also suggests that despite a lack of participant randomization, the IG and CG were similar with respect to their gender, ethnicity, and relationship status (*p* > 0.05), but not with respect to their age, education, employment status, or housing status (*p* < 0.001).

Table 2 indicates that the IG mean scores on the PSS, GAD-7, and PHQ-9 as well as the CMH score were significantly lower than scores for the CG. The mean scores on the PSS, GAD-7, and PHQ-9 scales and the CMH score were higher for the CG compared to the IG, 14.5%, 27.4%, 28.8%, and 20.9%, respectively.

Table 3 indicates that there were statistically significant differences in prevalence rates for moderate/high stress, likely GAD, and likely MDD during the study period. Participants in the IG had significantly lower prevalence rates for moderate/high stress (78.8% vs. 88.0%), likely GAD (31.4% vs. 46.5%), likely MDD (36.8% vs. 52.1%), and suicidal ideation/thoughts of self-harm (16.9% vs. 26.6%) in the two weeks preceding data collection, compared to respondents in the CG, but not for disturbed sleep symptoms (*p* > 0.01). The effect size of the intervention on each of these clinical variables was small, but significant.

## 9. Logistic Regression

To assess the impact of the supportive text message intervention on the likelihood for respondents to present with moderate/high stress, likely GAD, likely MDD, disturbed sleep, and suicidal ideation/thoughts of self-harm in the two weeks preceding data collection, whilst controlling for demographic characteristics, we entered all seven characteristics in Table 1 and “treatment type” into logistic regression models. Table 4 summarizes the output from five separate logistic regression models predicting likelihood of clinical variables of interest in IG vs. CG.

For moderate/high stress, the full model (eight predictors) was significant, *x*^2^ (df = 23, *n* = 2777) = 194.82, *p* < 0.001, suggesting the model distinguished between respondents who reported moderate/high stress and others. The model explained between 8.7% (Cox and Snell R2) and 13.9% (Nagelkerke R2) of the variance and correctly classified 81.3% of all cases. Controlling for all demographic characteristics, “intervention arm” made a unique statistically significant contribution (Wald = 14.2, *p* < 0.001) to the likelihood for respondents to present with moderate/high stress. Respondents who received daily supportive TextM for six weeks (IG) were 0.56 times less likely to report moderate/high stress during the study period compared to respondents who had not received the daily TextM (CG), when all demographic variables were controlled for (OR = 0.56; 95% CI = 0.41–0.75), as shown in Table 4.

For likely GAD, the full model containing all eight predictor variables was significant, *x*^2^ (df = 23, *n* = 2777) = 225.23, *p* < 0.001, indicating distinction between respondents who had likely GAD and those who did not. The model explained between 10.6% (Cox and Snell R2) and 14.5% (Nagelkerke R2) of the variance and correctly classified 63.9% of all cases. Controlling for all demographic characteristics, the “intervention arm” made a unique statistically significant contribution (Wald = 27.63, *p* < 0.001) to the likelihood for respondents to meet the cut-off threshold for likely GAD. The IG was about 0.55 times less likely to meet the cut-off threshold for likely GAD during the study period compared to CG, when all demographic variables were controlled for (OR = 0.55; 95% CI = 0.44–0.68), as shown in Table 4.

For likely MDD, the full model containing all eight predictors was significant, *x*^2^ (df = 23, *n* = 2777) = 194.97, *p* < 0.001, implying the model was able to distinguish between respondents who had likely MDD versus those who did not. The model explained between 9% (Cox and Snell R2) and 12.2% (Nagelkerke R2) of the variance and correctly classified 58.6% of all cases. Controlling for all demographic characteristics, “intervention arm” made a unique statistically significant contribution (Wald = 22.37, *p* < 0.001) to the likelihood for respondents to present with likely MDD. Respondents in the IG were about 0.59 times less likely to meet the cut-off threshold for likely MDD during the study period compared to the CG, when all demographic variables were controlled for (OR = 0.50; 95% CI = 0.47–0.73) as shown in Table 4.

For suicidal ideation, the full model containing all eight predictors was significant, *x*^2^ (df = 23, *n* = 2777) = 209.13, *p* < 0.001, implying the model discriminated between respondents who had suicidal ideation or thoughts of self-harm in the two weeks preceding data collection and those who did not. The model explained between 9.7% (Cox and Snell R2) and 15.4% (Nagelkerke R2) of the variance and correctly classified 80.7% of all cases. Controlling for all demographic characteristics, “intervention arm” made a unique statistically significant contribution (Wald = 15.13, *p* < 0.001) to the likelihood for respondents to have had suicidal ideation or thoughts of self-harm in the preceding two weeks. Respondents in the IG were about 0.59 times less likely to have had suicidal ideation or thoughts of self-harm in the two weeks preceding data collection compared to the CG, when all demographic variables were controlled for (OR = 0.59; 95% CI = 0.45–0.77) as shown in Table 4.

For disturbed sleep, the full model containing all eight predictors was significant, *x*^2^ (df = 23, *n* = 2777) = 55.82, *p* < 0.001, implying the model could distinguish between respondents who had experienced disturbed sleep in the preceding two weeks and those who had not. Nevertheless, the model only explained between 2.7% (Cox and Snell R2) and 4.1% (Nagelkerke R2) of the variance and correctly classified 78.3% of all cases. For sleep disturbance, controlling for all demographic characteristics, “intervention arm” failed to (Wald = 3.49, *p* = 0.15) distinguish IG and CG on disturbed sleep in the preceding two weeks. Thus, respondents in the IG were no more and no less likely to have experienced disturbed sleep in the preceding two weeks during the study period compared to the CG (OR = 0.77; 95% CI = 0.60–1.01), as shown in Table 4.

## 10. Discussion

To our knowledge, this is the first examination of the short-term impact (six weeks) of a texting-based intervention aimed at alleviating pandemic-associated stress, generalized anxiety disorder (GAD), major depressive disorder (MDD), and suicidal propensity during COVID-19 in comparison to a control group. This study demonstrates the effectiveness of Text4Hope over six consecutive weeks on various psychological symptomatology, including stress, GAD, MDD, and suicidal ideation or thoughts of self-harm, but not for disturbed sleep symptoms. A related study which had no control group reported there were statistically significant reductions in the prevalence rates for clinically meaningful stress and anxiety as well as statistically significant reductions in mean scores on the PSS-10 and GAD-7 scales when comparing the baseline and sixth week assessments in subscribers of Text4Hope [19]. A second longer term study which also had no control group reported there were statistically significant reductions in the prevalence rates for clinically meaningful stress, anxiety and depression as well as statistically significant reductions in mean scores on the PSS-10, GAD-7, and PHQ-9 scales when comparing the baseline and third month assessments in subscribers of Text4Hope [20].

The current study which, even though non-randomized, reasonably controls for the mental health impacts of the changing COVID-19 infection rates and their related health, social, financial, and occupational disruptions to the lives of the two study populations and augments the previous study.

Overall, the five clinical parameters measured showed likely significantly higher prevalence rates in the CG, ranging from 9.2% for moderate/high stress symptoms to 15.3% for likely MDD symptoms, compared to the IG. All clinical parameters, except for stress symptoms, have improved by over 25% and the CMH score was over 20% higher in the CG compared to the IG. Due to a lack of randomization of study participants between the IGs and CGs, there were some between group demographic differences with the CG population being younger with a larger proportion of students. Consequently, this cohort reported lower educational attainment and were more likely to rent accommodations or live with their families, rather than owning homes. The regression model controlled for such differences and revealed similar results for all the clinical presentations under study. As such, lower scores in IG compared to CG in all clinical domains suggests the effectiveness of Text4Hope as an intervention to alleviate psychological symptoms.

During the COVID-19 pandemic, technology-based interfaces have been widely deployed in a number of health-related services, including tracking the spread of COVID-19 [25], gathering data related to public knowledge and behavior regarding the pandemic [26], or providing mental health support during the pandemic [27]. Similarly, Text4Hope was designed in response to the different psychiatric burdens that may result from, or be worsened by, the pandemic. Generally, TextM are popular and enjoy high rates of acceptability and satisfaction, where upwards of 80% of message recipients report a marked improvement in their overall mental wellbeing [10]. Clinical advantages of TextM platforms are supported in several medical fields, including mental health: employed as reminders, support, and self-monitoring of clinical symptoms [11]. In the context of affective disorders, TextM were successfully deployed to generate positive feelings in people living with depression and bipolar disorder [28,29]. In a randomized control trial, TextM significantly reduced participant scores on the Beck Depression Inventory-II scale after three months of receiving twice-daily supportive TextM, compared to a CG, mean (SD) = 8.5 (8.0) vs. 16.7 (10.3), F (1, 49) = 9.54, *p* = 0.003, respectively [12]. Similar results were obtained when TextM were coupled with CBT; TextM were used to improve treatment adherence and track the progression of the clinical condition in patients with mood disorder [26]. Within the comorbidity of alcohol dependence and substance use disorder with depression, or either alone, TextM were recognized to enhance medication adherence, time to first drink, and relapse prevention [30,31].

Compared to the CG, Text4Hope improved GAD-7 and PSS scores in the IG; this suggests effects comparable to other computerized, web-based, or mobile based intervention programs that target anxiety and stress symptoms. Such programs usually express high initial rates of acceptance and feasibility by both therapists and patients [32]. A web-based CBT program was provided to graduate students in the United States for four-weekly sessions [33]. The program improved anxiety symptoms in 18.8% of the service recipients. In the same context, when combining a telephone service with computerized CBT, a reduction of 1.18 in GAD-7 mean score was observed [34], which is less than the 2.07 reduction in GAD-7 mean score for the IG, compared to the CG, as observed in our study. Additionally, this difference is comparable with the effect of some medications used to manage anxiety, such as sertraline. A United Kingdom study showed sertraline, when used for a six-week period, could reduce GAD-7 scores by (21%) [35], compared to (27.4%) in our study, albeit this difference was between the two treatment groups, rather than one group.

Improvement in stress symptoms scores in our study was inconsistent with another study in Japan in which an online CBT program of six to eight sessions was offered to university students [32]. In this study, the program impacted how clients thought about anxiety and stress, however, there were only reductions in anxiety symptoms [36]. Opposite to the “SleepTrackTXT2 behavioral intervention” that improved sleepiness, fatigue, and concentration among emergency medical service clinicians in the short-term [37], our study showed no significant change in the disturbed sleep symptoms in IG compared to the CG.

TextM are perceived as an acceptable, feasible, and supportive tool during the transition period after discharge for an attempted suicide [12,38]. A mobile (smartphone) app used for data collection in one study showed reliability (*r =* 0.84) when compared with paper-based PHQ-9 scores [39]. As such, text messages and online applications can be reliably used to assess the mental health impacts of a population during disasters. Text4Hope yielded lower scores of suicidal thoughts in the IG compared to the CG. Taken together, texting programs appear to improve positive thinking and reduce negative suicidal/self-harm thoughts, especially during major crises.

The study has several limitations and therefore our results should be interpreted with caution. First, the two groups under study were not randomly assigned, which was reflected in the significant differences observed in the demographic characteristics between the IG and CG. Efforts were made to control for these differences in the logistic regression model used in the analysis. Second, effect sizes were relatively small. However, interventions that do not include therapist support often report low effect sizes compared to those including therapists [33,40]. Third, the opt-out rate at three months from Text4Hope is high (around 26%) and so it is possible that the impact of the intervention could be different in the group who unsubscribe from the program. In a review of 93 mental health apps targeting anxiety, depression, or emotional well-being, the medians of app 15-day and 30-day retention rates were only 3.9% (IQR 10.3%) and 3.3% (IQR 6.2%), respectively [41]. This indicates that ourText4Hope program achieved a higher retention rate compared to other mental health apps. This may be because Text4Hope is unidirectional and requires no additional effort or action on the part of the subscriber following enrolment. It is also possible that the message content, crafted by mental health professionals, the high anxiety, stress, and depression levels experienced by the population level due to the COVID-19 pandemic, and the reduced availability of face-to face services contributed to the high Text4Hope retention rate. Fourth, although we used validated instruments to assess stress, anxiety, and depression, these self-assessed instruments are not diagnostic of clinical conditions for which structured clinical interviews are needed. Finally, the majority of respondents in our study identified as female, 26 to 60 years of age, partnered, and employed. Thus, it is unclear how these results would generalize to a broader population of Canadians with different sociodemographic characteristics. However, the baseline demographic and clinical characteristics of the sample are similar to the characteristics of the larger sample of subscribers who completed baseline surveys within the first three months following the launch of Text4Hope [42]. It would therefore be reasonable to conclude that the supportive messages would have similar effects on all Text4Hope subscribers. Notwithstanding the limitations, our study achieved very high power. Based on the actual sample size of 555 minimum per group achieved in our study and the mean CMH scores observed for the IG and CG, our study achieved a power of 1.0.

## 11. Conclusions

Texting-based programs are evidently feasible, cost-effective, and of clinical significance. They can be deployed quickly during pandemics to support at risk populations, which can be crucial to mitigating negative short- and long-term psychological impacts. After six weeks of its application, the Text4Hope program effectively ameliorated various psychiatric burdens during the COVID-19 pandemic, including stress, GAD, MDD, and thoughts of suicide/self-harm. These negative thoughts and feelings are usually triggered and easily thrive, during major crises and natural disasters. To this end, similar initiatives might be considered, especially in the context of technology-based, remotely accessible, and population-level interventions, within different clinical contexts, for vulnerable populations. This study along with two related published outcome studies [16,17] on the Text4Hope program may serve to provide evidence-based support for such policy implementation in high-, middle-, and low-income countries. The research team therefore plans to explore national scale-up and implementation of the Text4Hope program in multiple languages to benefit all Canadians. The team will also disseminate this program for adaptation and potential global use through the E-Text4PositiveMentalHealth platform, currently under development, and formation of partnerships with national and regional health authorities and institutions.

## Figures and Tables

**Table 1:** Demographic characteristics of study participants.

Demographic Characteristics	Intervention Group (IG) *n* = 2011 *	Control Group (CG) *n* = 756 *	*p*-Value **	Chi-Square	Degrees of Freedom (df)	Total *n* (%)
**Gender**						
Male	214 (10.7)	72 (10.9)	0.96	0.082	2	286 (10.7)
Female	1767 (88.1)	580 (87.7)				2347 (88.0)
Other Gender	25 (1.2)	9 (1.4)				34 (1.3)
**Age (Years)**						
≤25	173 (8.6)	92 (14.2)				265 (10.0)
26–40	554 (27.6)	191 (29.5)	<0.001	20.39	3	745 (28.1)
41–60	1002 (49.9)	289 (44.6)				1291 (48.6)
>60	278 (13.9)	76 (11.7)				354 (13.3)
**Ethnicity**						
Caucasian	1669 (83.6)	529 (80.6)				2198 (82.8)
Indigenous	59 (3.0)	29 (4.4)	0.17	5.09	3	88 (3.3)
Asian	105 (5.3)	34 (5.2)				139 (5.2)
Other	164 (8.2)	64 (9.8)				228 (8.6)
**Education**						
Less than High School Diploma						
High School Diploma	44 (2.6)	43 (6.5)				87 (3.7)
Post-Secondary	116 (6.9)	65 (9.8)	<0.001	34.06	3	181 (7.7)
Other Education	1512 (89.9)	542 (82.0)				2054 (87.7)
	10 (0.6)	11 (1.7)				21 (0.9)
**Relationship status**						
Married/Cohabiting/Partnered	1100 (65.4)	453 (68.5)				1553 (66.3)
Separated/Divorced	171 (10.2)	52 (7.9)	0.25	5.39	4	223 (9.5)
Widowed	41 (2.4)	10 (1.5)				51 (2.2)
Single	354 (21.0)	138 (20.9)				492 (21.0)
Other	17 (1.0)	8 (1.2)				25 (1.1)
**Employment**						
Employed	1185 (71.1)	453 (68.5)				1638 (70.4)
Unemployed	203 (12.2)	52 (7.9)	<0.001	186.86	4	255 (11.0)
Retired	173 (10.4)	10 (1.5)				183 (7.9)
Student	80 (4.8)	138 (20.9)				218 (9.4)
Other	26 (1.6)	8 (1.2)				34 (1.5)
**Housing Status**						
Own Home	1160 (69.6)	400 (61.9)				1560 (67.5)
Living with Family	150 (9.0)	88 (13.6)	<0.001	18.59	3	238 (10.3)
Renting	343 (20.6)	147 (22.8)				490 (21.2)
Other	13 (0.8)	11 (1.7)				24 (1.0)

* There was no imputation for missing values for a particular characteristic and so total number of responses for each demographic variable is less than the Total *n* for the Intervention Group (IG) or Control Group (CG). ** Bonferroni corrected significant *p* < 0.007.

**Table 2:** Independent sample *t*-test comparing the mean scores for IG and CG on the Perceived Stress Scale (PSS), the Generalized Anxiety Disorder 7-item (GAD-7), and Patient Health Questionnaire-9 (PHQ-9) scales and the Composite Mental Health (CMH) score.

		*n*	Mean	Std. Deviation	Std. Error	T	df	*p*-Value *	Mean Difference (MD)	95% Confidence Interval of MD
**PSS Total Score**	IG	1864	19.50	7.12	0.16	8.41	2472	<0.001	2.82	2.17–3.48
	CG	610	22.32	7.41	0.30					
**GAD-7 Total Score**	IG	1704	7.55	5.31	0.13	7.70	2308	<0.001	2.07	1.54–2.60
	CG	557	9.62	6.08	0.26					
**PHQ-9 Total Score**	IG	1738	8.60	5.98	0.14	8.33	2259	<0.001	2.48	1.86–3.10
	CG	572	11.08	6.73	0.28					
**CMH Score**	IG	1700	35.64	16.94	0.41	8.77	2253	<0.001	7.44	5.78–9.12
	CG	555	43.08	18.55	0.78					

* Bonferroni corrected significant *p* < 0.0125.

**Table 3:** Chi-square test of association between prevalence of clinical parameters and study arm.

	Study Arm	
	IG *n* (%)	CG *n* (%)
**Perceived Stress**		
Moderate/High Stress ^a^	1468 (78.8%)	537 (88.0%)
*p*-value	<0.001 *	
Effect Size (Phi)	−0.102	
**Generalized Anxiety Disorder (GAD)**		
GAD likely ^b^	535 (31.4%)	265 (46.5%)
*p*-value	<0.001 *	
Effect Size (Phi)	−0.146	
**Major Depressive Disorder (MDD)**		
MDD likely ^c^	639 (36.8%)	298 (52.1%)
*p*-value	<0.001 *	
Effect Size (Phi)	−0.135	
**Suicidal Ideation/Thoughts of Self Harm ^d^**		
Experienced Suicidal Ideation/Self Harm Thoughts	293 (16.9%)	152 (26.6%)
*p*-value	<0.001 *	
Effect Size (Phi)	−0.106	
**Sleep Disturbances ^e^**		
Experienced Sleep Disturbances	1336 (76.9%)	466 (85.1%)
*p*-value	0.020	
Effect Size (Phi)	−0.047	

^a^ Moderate/High Stress defined as PSS ≥ 14. ^b^ Likely GAD defined as GAD-7 ≥ 10. ^c^ Likely MDD defined as PHQ-9 ≥ 10. ^d^ Suicidal ideation/thoughts of self-harm defined as PHQ-9 item 9 ≥ 1. **^e^** Sleep Disturbances defined as PHQ-9 item 3 ≥ 1. * Bonferroni corrected significant *p* < 0.01.

**Table 4:** Odds for subscribers in the IG to have various clinical characteristic compared to the CG.

Clinical Variables of Interest	*p*-Value	Odds Ratio	95% CI for OR	
			Lower	Upper
Moderate/High Stress^a^	<0.001	0.56	0.41	0.75
GAD likely ^b^	<0.001	0.55	0.44	0.68
MDD likely ^c^	<0.001	0.50	0.47	0.73
Experienced Suicidal Ideation/Self Harm Thoughts	<0.001	0.59	0.45	0.77
Experienced Sleep Disturbances	*0.150*	0.77	0.60	1.01

^a^ Moderate or High Stress defined as PSS ≥ 14 ^b^ Likely GAD defined as GAD-7 ≥ 10 ^c^ Likely MDD defined as PHQ-9 ≥ 10.

## Data Availability

Data for this study is available and can be released following reasonable request by writing to the corresponding author.
